# Prolonged Duration of Viral Shedding of SARS-CoV-2: A Case Report

**DOI:** 10.5811/cpcem.2020.7.49005

**Published:** 2020-08-08

**Authors:** Brandon Fong, Kory S. London

**Affiliations:** Thomas Jefferson University Hospital, Department of Emergency Medicine, Philadelphia, Pennsylvania

**Keywords:** SARS-CoV-2, hypoxemic, respiratory failure, cardiogenic shock

## Abstract

**Introduction:**

The literature on the clinical course of severe acute respiratory syndrome coronavirus 2 (SARS-COV-2) suggests patients continue shedding viral particles typically for an average of 20 days until the body builds immunity against the infection. However, a few cases have shown prolonged duration in viral shedding and highlight the significant increased mortality in these patients. It has also been suggested that multiple strains of SARS-COV-2 exist, keying the possibility to reinfection.

**Case Report:**

We present a case of a 57-year-old male who presented twice over 37 days with symptoms related to SARS-COV-2, and only on his second visit was found to be in hypoxemic respiratory failure and cardiogenic shock. He also reportedly had a period of convalescence in between presentations.

**Discussion:**

This case highlights the still unclear disease course of SARS-COV-2 and the need for diligence in providing strong follow-up instructions and evaluation for sequelae of the infection.

## INTRODUCTION

The novel severe acute respiratory syndrome coronavirus 2 (SARS-COV-2) outbreak has been shown to cause respiratory failure, cytokine storm, and disease complications such as thrombosis.^1^–^4^ The immune system takes around three weeks to develop antibodies to combat the infection and enter into a period of convalescence. This is consistent with prior studies that reported the median duration of viral shedding to be 20 days.^2^ However, one case report and retrospective study documented in China noted prolonged shedding of viral ribonucleic acid (RNA) as detected by reverse transcription-polymerase chain reaction (RT-PCR).^6^ Finally, a single case of a patient with an initially severe course of SARS-COV-2 was readmitted nearly two months later after a mild recurrence of symptoms.^5^ None of these patients developed severe symptoms late in their course. Herein we present a case of a patient who presented initially with mild symptoms of SARS-COV-2 and was later readmitted with a fulminant course. This case challenges the understanding of viral immunity and progression of this novel disease.

## CASE REPORT

**Image 1:**
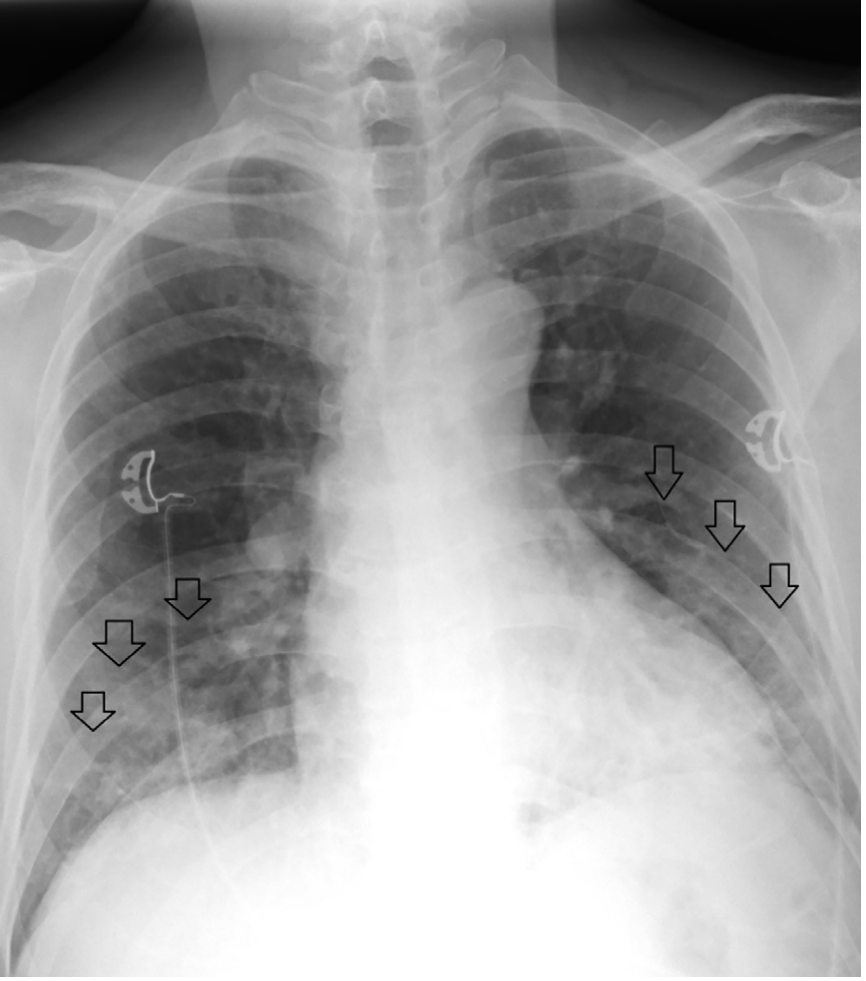
Initial anterior-posterior chest radiograph of a patient with coronavirus disease 2019 demonstrating bilateral lower lobe infiltrates (arrows).

A 57-year-old male with a past medical history of hypertension, type 2 diabetes mellitus, coronary artery disease with history of non-ST elevation myocardial infarction in February 2019, and ischemic cardiomyopathy with reduced ejection fraction of 40% initially presented to the emergency department in mid-April with symptoms of subjective fever, cough, and mild chest pain for four days. He tested positive for SARS-CoV-2 via RT-PCR, along with a one-view chest radiograph (CXR) interpreted as multifocal pneumonia of bilateral lower lobes (Image 1).

His electrocardiogram (ECG) showed normal sinus rhythm along with unchanged infero-lateral ST depression from prior ECGs. His vital signs were within normal limits. He was observed for one day and discharged with self-isolation precautions. After discussion with his roommates and landlord, we learned that his symptoms had improved in the intervening period but never resolved.

In mid-May he was brought in by emergency medical services for fatigue and respiratory distress, having worsened over the previous 48 hours. His triage vital signs were oral temperature of 97.4º Fahrenheit, heart rate 140 beats per minute, blood pressure 73/32 millimeters of mercury (mmHg), respiratory rate of 26 breaths per minute, and pulse oximetry of 63% on room air. He was placed on bilevel positive airway pressure and subsequently became obtunded. He went into pulseless electrical activity (PEA), coded with return of spontaneous circulation after 20 minutes, and was intubated. Physical examination was remarkable for crackles in bilateral bases of the lungs and cold distal upper and lower extremities. ECG was non-diagnostic but showed sinus tachycardia without ST segment elevation. Point-of-care ultrasound showed a severely depressed ejection fraction without evidence of pulmonary embolism/hypertension.

**Image 2:**
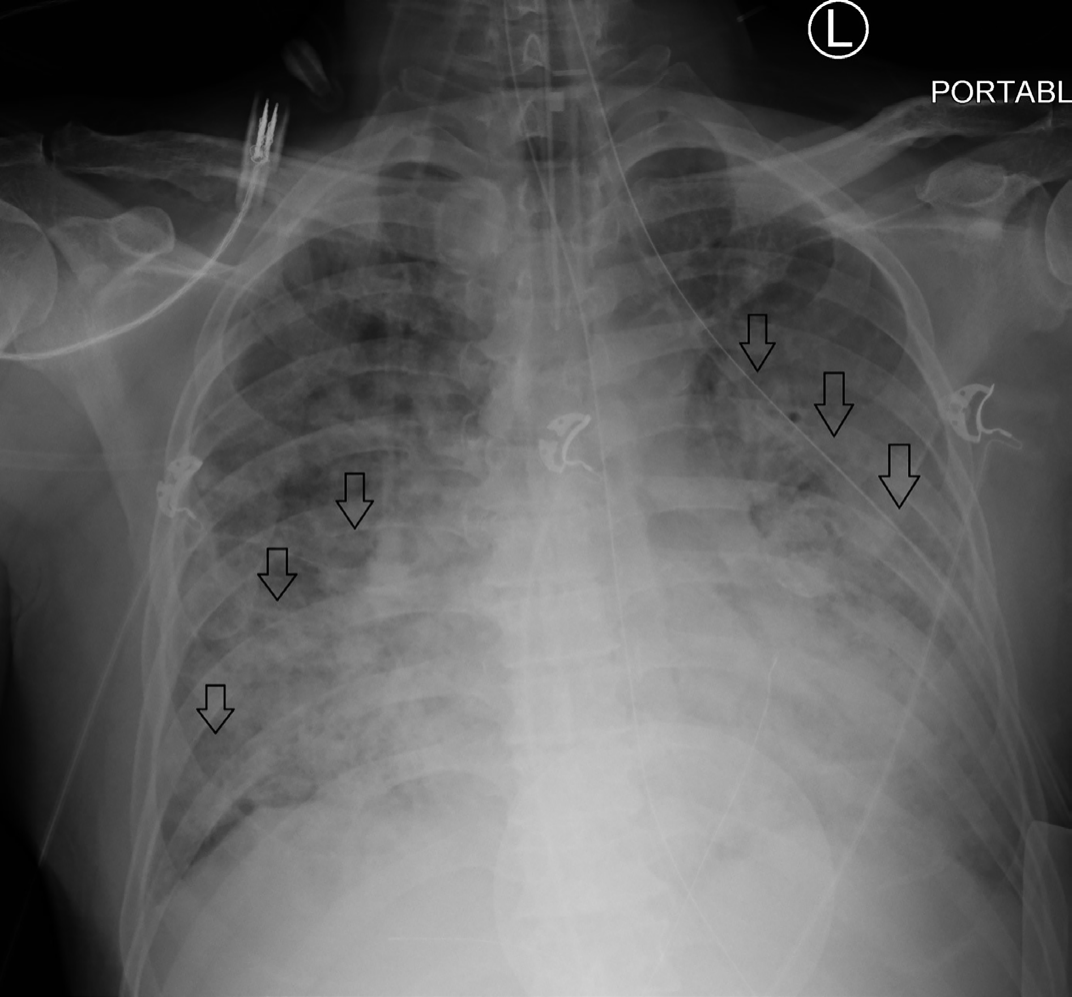
Anterior-posterior chest radiograph (CXR) 37 days after the CXR shown in Image 1 in a patient with coronavirus disease 2019, demonstrating markedly worsening bilateral pulmonary infiltrates (arrows).

Significant laboratory results were as follows: repeat SARS-CoV-2 positive; white blood cells 12.3 thousand per cubic millimeter (K/mm^3^) (reference range 4.5–11.5 K/mm^3^); lactic acid was 15 millimoles per liter (mmol/L) (reference range 0.5–2.2 mmol/L); troponin 889 nanograms per liter (ng/L) (reference range <6 ng/L); creatinine 1.75 milligrams per deciliter (mg/dL) (reference range 0.84–1.21 mg/dL); venous blood gas pH 6.89 (reference range 7.32–7.43) and partial pressure of carbon dioxide (pCO_2_) of 81 mmHG (reference range 38–50 mmHg); D-dimer 2735 nanograms per milliliter (reference range <250 ng/mL). He was human immunodeficiency virus negative with no prior history of liver dysfunction. A one-view CXR was interpreted as multifocal pneumonia, but with markedly worsening bilateral pulmonary infiltrates compared to his previous CXR from his prior visit (Image 2).

CPC-EM Capsule

What do we already know about this clinical entity?

*While the novel coronavirus disease 2019 has a median viral shedding course of 20 days, one study reported prolonged viral shedding greater than 20 days*.

What makes this presentation of disease reportable?

*Our patient was seen and treated twice, 37 days apart, with a period of convalescence intervening, and suffered a fulminant and fatal disease course*.

What is the major learning point?

*The case highlights the still unclear disease course. Patients suffer many sequelae, but primary severe acute respiratory coronavirus 2 is also a highly morbid disease*.

How might this improve emergency medicine practice?

*This case shows importance of educating patients about return precautions and for adequate follow-up care to monitor progression of symptoms after 20 days*.

He was admitted to the medical intensive care unit (MICU) for hypoxemic respiratory failure with mixed picture of cardiogenic shock and septic shock and was started on vancomycin, piperacillin/tazobactam, and azithromycin for broad-spectrum antimicrobial coverage. In the MICU, the patient continued to have low mean arterial pressures despite being on multiple vasopressor medications: norepinephrine; vasopressin; and epinephrine. He was started on dexamethasone for concern for adrenal insufficiency. He developed acute renal failure and ischemic hepatitis with elevated creatinine to 4.89 mg/dL; alanine transaminase 6666 units per liter (U/L) (reference range 7–55 U/L); and aspartate aminotransferase of 7115 U/L (reference range 8–48 U/L), respectively. On his third day of hospitalization, he went into PEA again and finally into asystole and was not revivable. His blood, urine, and sputum cultures were ultimately negative.

## DISCUSSION

This case raises the possibility that patients may suffer cardiopulmonary compromise long after their initial presentation of SARS-COV-2, or may be prone to re-infection. One study reported a median duration of viral shedding of 20.0 days (interquartile range [17.0–24.0]) following symptom onset.^1^ These patients developed acute respiratory distress syndrome between 8–12 days following onset of symptoms. Moreover, another report from China showed, in a cohort of 41 patients, the duration of RNA shedding ranged from 18–48 days.^2^ These patients also had severe illness at their index visits, followed by convalescence with persistent shedding of the virus. One final case report from China documented how a patient who developed acute respiratory distress from SARS-CoV-2 12 days into their illness, had persistent viral shedding in the respiratory tract for 46 days from illness onset.^6^ None of these patients had a similar course to our patient, who suffered a very late fulminant course with respiratory failure and shock 37 days following onset of symptoms.

Recently, a study of convalescent serum revealed that many patients do not develop high levels of neutralizing antibody activity.^7^ This finding leaves unclear the issue of duration of immunity post-infection. Similarly, it has been shown that there is significant genetic variation in SARS-COV-2, with the possibility of coexisting viral strains in some communities.^8^ This posits the possibility that patients may become re-infected with different SARS-COV-2 strains and, ergo, prior infection may not necessarily be protective. While specific genetic serotyping of the patient was not available, given the very long duration between initial symptoms and decompensation, this suggests the possibility that he may have suffered two distinct illnesses.

There are certain risk factors that place patients at a higher likelihood of susceptibility to prolonged viral shedding and complications. One study reported these risk factors included male gender, being elderly, and having hypertension, along with mechanical ventilation and corticosteroid use; however, the study data did not incorporate patients past duration of RNA shedding after 22 days.^9^ Our patient had two of these risk factors (male and concomitant hypertension) reinforcing his chances of prolonged viral shedding. Additionally, he also had a complex past medical history, having suffered a recent myocardial infarction one year prior to his death. Known coronary artery disease is an especially troubling risk factor, with dramatically increased rates of morbidity and mortality.^3^,^10^ Our case reiterates this finding with our patient who had extensive cardiovascular comorbidities, thus increasing his predisposition to cardiac complications resulting in his cardiogenic shock.

## CONCLUSION

In this case, a patient returned five weeks following mild SARS-COV-2 infection with a fulminant course. Increased attention to those with significant comorbidities and providing strict and accurate follow up information to patients is essential given the unknowns associated with this novel disorder.
